# The Value of Serum Cell-Free DNA Levels in Patients With Schizophrenia

**DOI:** 10.3389/fpsyt.2021.637789

**Published:** 2021-03-30

**Authors:** Ling-yun Chen, Jing Qi, Hong-lei Xu, Xiang-yun Lin, Ya-jun Sun, Shao-qing Ju

**Affiliations:** ^1^Center of Laboratory Medicine, Nantong Mental Health Center, Nantong, China; ^2^Reaserch Center of Clinical Medicine, Affiliated Hospital of Nantong University, Nantong, China; ^3^Neurology Laboratory, Affiliated Hospital of Nantong University, Nantong, China; ^4^School of Public Health, Nantong University, Nantong, China; ^5^Center of Laboratory Medicine, Affiliated Hospital of Nantong University, Nantong, China

**Keywords:** biomarker, schizophrenia, apoptosis, circulating cell-free DNA, *Alu*

## Abstract

**Background:** Schizophrenia is a severe mental disorder, which has a major impact on the quality of life and imposes a huge burden on the family. However, the pathogenesis of schizophrenia remains unclear and there are no specific biomarkers. Therefore, we intend to explore whether cf-DNA levels are related to the occurrence and development of schizophrenia.

**Methods:** We analyzed and compared the concentration of cf-DNA in 174 SZ patients and 100 matched healthy controls by using quantitative real-time PCR by amplifying the *Alu* repeats.

**Results:** We found that cf-DNA levels in peripheral blood reliably distinguished SZ patients from healthy controls (*P* < 0.05). The ROC analysis also supports the above conclusion. By tracking the absolute concentration of serum cf-DNA in primary cases, we found a distinct increase before treatment with antipsychotics, which decreased progressively after treatment.

**Conclusions:** The present work indicates that cf-DNA may improve the efficiency of disease diagnosis, and the level of cf-DNA plays a predictive role in the development of schizophrenia. By evaluating the level of cf-DNA, we might play a certain role in a more reasonable and standardized clinical treatment of schizophrenia.

## Introduction

Schizophrenia (SZ) is a major public problem that impairs brain function and affects approximately 1% of the world population (1–3). Different from many other diseases, the exact etiology and pathogenesis of SZ remains elusive. Diagnoses are still determined by the medical history together with the mental symptoms and disease progression. To date, no definite laboratory examination or laboratory test has been demonstrated to support an accurate clinical diagnosis. Such diagnoses are subject to subjective factors and higher requirements for doctor's clinical experience. As potential biomarkers and therapeutic targets have yet to be established (4), the development of biomarkers to improve diagnosis and prognosis is an urgent task.

In the past, hypotheses regarding the causes of SZ have been proposed, including abnormal neurotransmission, nerve development and generation, immune system disorders and neuronal apoptosis (5). Moreover, apoptosis is a physiological process of cell death and has been confirmed to be involved in the pathological process of SZ. Pro-apoptotic stimulation such as inflammation, autoimmunity, and oxidative stress may lead to the accumulation of reactive oxygen species (ROS) in mitochondria, affect the oxidative metabolic process, and then trigger the apoptosis of neurons and glial cells (6–8). Early studies have shown that 20–80% of neurons in the central nervous system die from apoptosis (9) and have reported the aberration of apoptotic regulatory proteins and DNA fragmentation status in the posterior temporal cortex (10, 11). These studies laid out the foundation for exploring the potential role of apoptosis in the pathogenesis of SZ.

Cf-DNA was first observed by French scientists Mandel and Metais in human plasma in 1,948 (12). Since then, many scholars have made in-depth studies on the content of cf-DNA in the blood of cancer patients (13). *Alu* is a cell-free DNA molecule that can be easily detected. The *Alu* sequence is a rich intermediate repeat sequence unique to the primate genome and is the largest family with short scattered repeat sequences with a high homology CG (70–98%). Now, it is generally believed that cf-DNA, a type of cell-free extracellular DNA, is mainly derived from apoptosis and necrosis (14–16). Using PCR, Wang (17) found that long DNA fragments associated with necrosis could be distinguished from short DNA fragments generated by physiological apoptosis. Research shows that cerebrospinal fluid cell-free mitochondrial DNA is associated with mild neurocognitive impairment (18, 19). Studies have shown that most of the factors released by activated microglia cells in SZ patients have toxic effects on neurons (20). Evidence suggests that antipsychotics exhibit neuroprotective potential (21), can inhibit inflammatory responses, combat neuronal damage, and reduce cell apoptosis and damage (22–24).

In our study, we investigated the serum level of *Alu* in SZ patients and explored its clinical value as a serum auxiliary biomarker for SZ. We found that the *Alu* content in the SZ group was significantly higher than that in the control group. Furthermore, *Alu* was not correlated with gender, age at onset, current age, duration of psychosis, current treatment or family history. By trcking the 57 newly diagnosed SZ patients, we found the level of *Alu* before treatment was higher than the level at post treatment. This study presents basic research on disease surveillance and may lay a foundation for the pathogenesis of SZ and the search for potential biomarkers.

## Materials and Methods

### Subjects

Serum samples were collected from 174 SZ patients (the SZ group) from the Nantong Mental Health Center between December 1st 2017 and August 1st 2018. The SZ patients were divided into the following subtypes: undifferentiated (*n* = 132), paranoid (*n* = 11), acute SZ-like psychosis (*n* = 29), and other subtypes (*n* = 2). The diagnosis of SZ was based on the criteria of the Diagnostic and Statistical Manual of Mental Disorders, 4th Edition, as well as the International classification of diseases, 10th Edition.

During the same period, 100 healthy controls (the HC group) were selected from the Physical Examination Center of the Affiliated Hospital of Nantong University (Nantong, China) who had no history of autoimmune diseases, tissue injuries or traumas and whose hematological-biochemical profiles were normal at the time of examination. The demographic details of all the participants are shown in Table 1.

**Table 1:** Demographic details of participants.

	**Healthy individuals**	**SZ patients**	**χ^2^**	***P*-value**
Total Number (*N*)	100	174		
Sex			0.567	0.451
M	47	90		
F	53	84		
Age (year)			0.207	0.649
<25	46	85		
≥25	54	89		

*P > 0.05 was considered not statistically significant*.

This study was approved by Nantong Forth People's Hospital ethics committee (2019 K007) and all participants signed an informed consent form.

### Clinical Assessment

We used the Nottingham Onset Schedule (25) to record the onset date of the psychotic symptoms and the beginning of pharmacological treatment. Pharmacological treatment duration was defined by the period (in weeks) between the beginning of pharmacological treatment and blood collection. Finally, the interval (in weeks) from the first manifestation of psychotic symptoms to blood collection defined the total duration of the metal disorder.

The age at the onset of symptoms was collected for all SZ patients. In addition, the positive and negative syndrome scale (26) was used for all participants.

### Plasma Collection and cf-DNA Isolation

Blood samples were collected in separating gel vacuum collection tubes and centrifuged at 3000 × g for 10 min. The upper-layer supernatant was immediately stored in an RNase-free Eppendorf tube at −80°C until use. Cf-DNA was extracted from 0.2 ml of serum using the TIANLONG DNA Kit (Suzhou, China) according to the manufacturer's protocol.

### Quantitative Real-Time Polymerase Chain Reaction (RT-qPCR)

RT-qPCR was performed with Applied Biosystems 7,500 (Applied Biosystems, CA, USA). We performed experiments with absolute quantification to verify the results. The target for RT-qPCR was the consensus sequence of human *Alu*-interspersed repeats: a 115 bp Alu amplicon (*Alu115*) that represents both shorter and longer cf-DNA fragments. The diluted cf-DNA template was used with 1 × FastStart Universal SYBR GreenImaster mix (Roche, Switzerland) for RT-qPCR, which was performed by a Real-Time PCR System (Life Technologies, USA) at 95°C for 10 min, followed by 35 cycles of denaturation at 95°C for 15 s and annealing at 64°C for 1 min (27). The absolute level of *Alu* was quantified using the standard curve (from 0.222 to 22,200 ng/ml) of human genome DNA (Promega, Madison, WI, USA). Mean values were calculated from triplicate reactions.

### Detection of Biochemical Indicators

The liver function, kidney function, blood glucose, electrolytes, cardiac enzyme spectrum, and lipid profile of 174 patients were detected on an Olympus AU680 biochemical analyzer.

### Statistical Analysis

The concentration of *Alu* was depicted as the median and 25th and 75th percentiles. Statistical analysis was performed using the SPSS software package version 20.0 (SPSS Inc., Chicago, USA). Statistical significance was tested by the Mann-Whitney unpaired test analysis of variance or the Chi-square test, as appropriate. *P* < 0.05 was considered statistically significant. Receiver operating characteristic (ROC) curves and area under the ROC curve (AUC) were used to assess the diagnostic value. The maximum AUC area was determined by the specificity and sensitivity of the optimal cut-off point. The figures were partially drawn by GraphPad Prism 5.0 software (Graphpad Software Inc., CA, USA).

## Results

### The Level of *Alu* in SZ Patients

**Figure 1:**
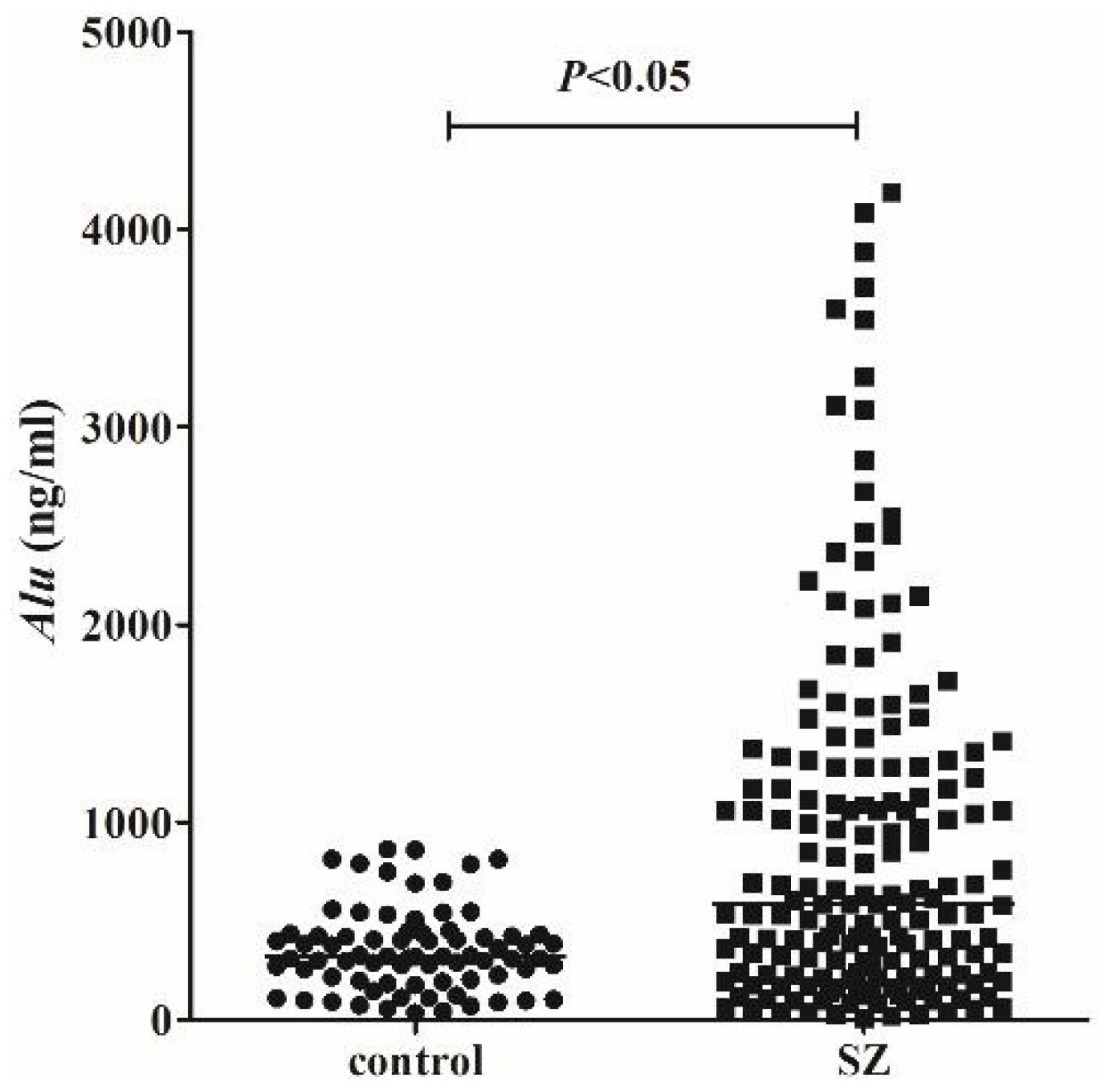
cf-DNA levels measured by RT-qPCR. Statistical comparisons were performed using logistic analysis. When *P*-value <0.05, the difference was statistically significant. The horizontal line represents the median for each group.

**Figure 2:**
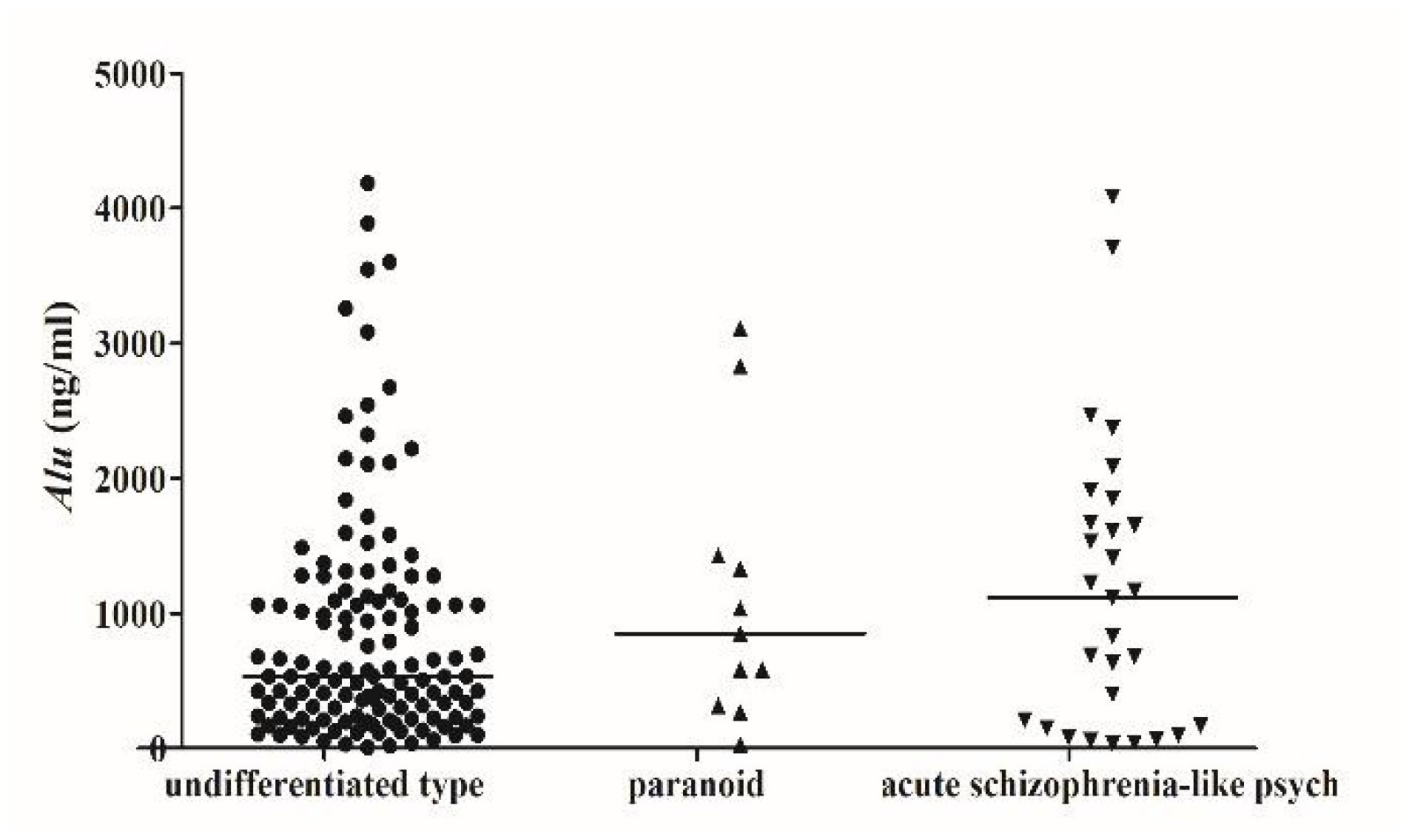
Scatter plot of serum Alu from patients with different types of schizophrenia. SZ patients were divided into undifferentiated type (132), paranoid (11), acute schizophrenia-like psychosis (29), and other subtypes (2), no statistical differences between different classification groups (*P* > 0.05).

We detected *Alu* using RT-qPCR of 115 bp amplicons. The concentration of *Alu* in the SZ group [586.0 ng/ml (interquartile range: 228.4–1275.7 ng/ml)] was significantly (*P* < 0.05) higher than that of the HC group [318.3 ng/ml (253.5–427.4 ng/ml)] (Figure 1). The concentration of *Alu* exhibited no statistical differences (*P* > *0.05*) between the different subtypes, which were as follows: undifferentiated [533.5 ng/ml (237.1–1095.9 ng/ml)], paranoid [849.9 ng/ml (450.5–1378.6 ng/ml)], and acute SZ-like psychosis [1115.3 ng/ml (167.1–1673.4 ng/ml)] (Figure 2).

### Diagnostic Utility of Serum *Alu* in SZ Patients

**Figure 3:**
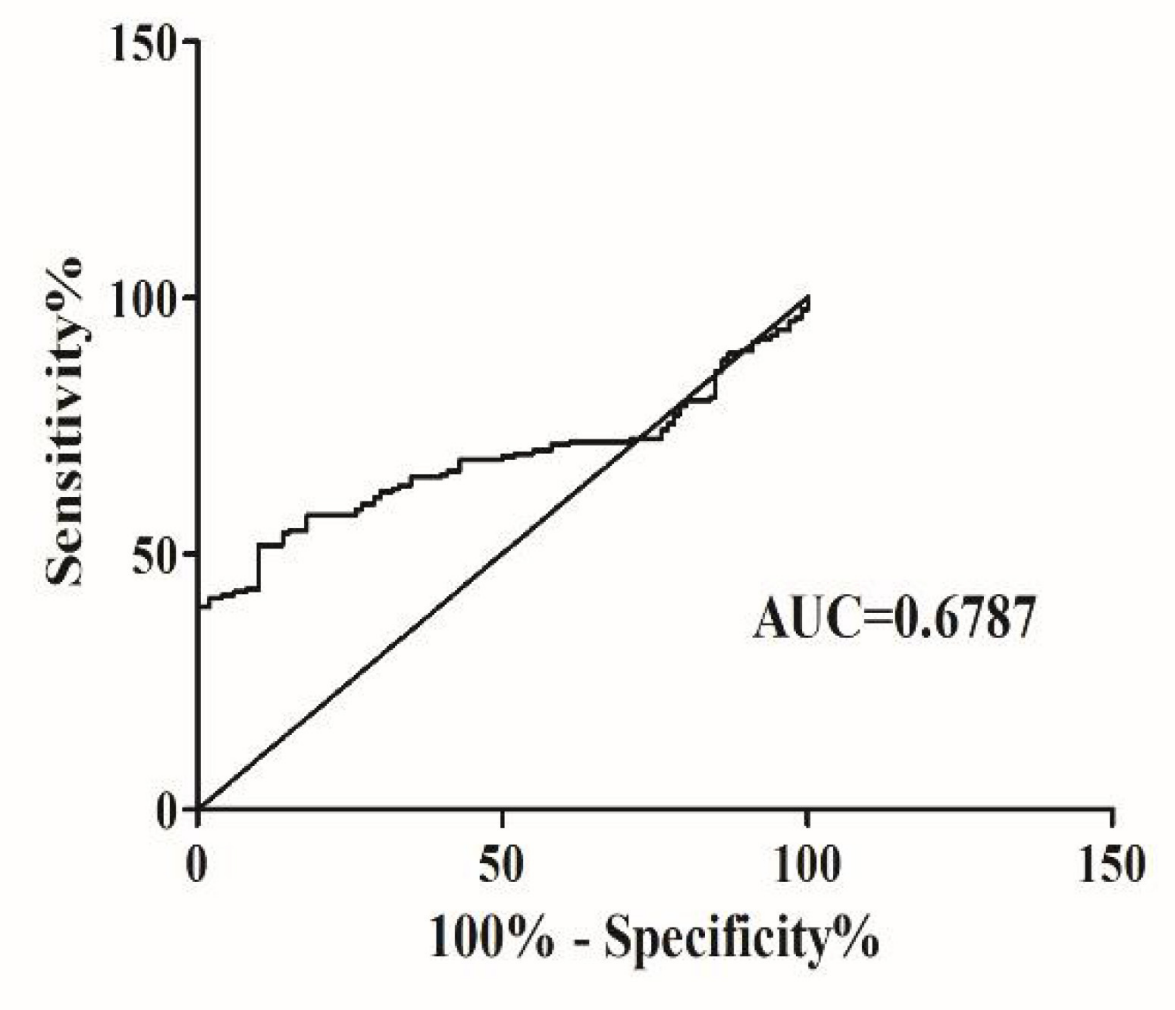
The ROC analysis of Alu between SZ patients and healthy controls. RT-qPCR and ROC curve analysis for predicting cf-DNA as a SZ diagnosis biomarker. The area under the curve (AUC) value was 0.6787.

To investigate the characteristics of serum *Alu* as a potential marker for SZ, ROC curves were constructed on data from all participants, including 174 SZ patients and 100 healthy controls. Serum *Alu* effectively differentiated SZ patients from normal controls with an AUC of 0.6787, with a 95% confidence interval of 61.66–74. 08% (Figure 3). When the cut-off value was 567.1 ng/ml, the sensitivity was 51.72% and the specificity was 90%, with a 95% confidence interval of 44.04–59.35%. Based on the cut-off value of 567.1 ng/ml, the Alu level exceeds the cut-off value in 47.7% undifferentiated type (63/132), 72.7% paranoid (8/11) and 65.6% acute SZ-like psychosis (19/29).

### The Correlation Between *Alu* and Clinical Feature in SZ Patients

We examined potential correlations between clinical features and the level of *Alu*. The analysis demonstrated no statistically significant correlation between cf-DNA levels and clinical variables, such as gender (*P* > 0.05), age at onset (*P* > 0.05), current age (*P* > 0.05), duration of psychosis (*P* > 0.05), current treatment (*P* > 0.05) and family history (*P* > 0.05) (Table 2).

**Table 2:** Clinical features of patients of SZ (*n* = 174) according to the specific diagnostic categories.

	***N***	***Alu* Median (interquartile range) (ng/ml)**	***P-*value**
Total	174	586.0 (228.4–1275.7)	
Gender			0.3816
Male	90 (51.7 %)	535.4 (228.4–1092.4)	
Female	84 (48.3 %)	625.0 (275.6–1415.6)	
Psychosis onset age (y)			0.9418
Up to 16	39 (22.4 %)	609.6 (256.9–1412.4)	
17 to 24	77 (44.3 %)	634.3 (296.6–1197.6)	
25 to 34	47 (27.0 %)	591.3 (220.2–1331.0)	
35 or more	11 (6.3 %)	872.0 (353.0–1251.1)	
Current age (*y*)			0.3299
13 to 24	85 (48.9 %)	776.7 (343.7–1430.1)	
25 to 34	60 (34.5 %)	631.8 (296.6–1113.4)	
35 or more	29 (16.7 %)	422.8 (210.5–1114.7)	
Duration of psychosis (in weeks)			0.2301
Up to 12	42 (24.1 %)	849.9 (318.2–1673.5)	
13 to 52	28 (16.1 %)	687.2 (346.8–2093.1)	
53 or more	104 (59.8 %)	576.7 (254.2–1113.4)	
Current treatment			0.6479
AP	133 (76.4 %)	650.6 (240.3–1326.4)	
AP+AD	19 (10.9 %)	671.1 (465.3–1601.1)	
AP+MS	22 (12.6 %)	767.9 (463.3–1156.0)	
Family history			0.4306
Yes	17 (9.8 %)	1015.1 (414.9–1279.0)	
No	157 (90.2 %)	584.5 (225.6–1275.5)	

*P < 0.05 was considered statistically significant*.

*SZ, schizophrenia; AP. anti-psychotics; AD, antidepressants; MS, mood stabilizers; CGI, clinical global impression*.

We also examined potential correlations between *Alu* and the scores of SZ patients. The analysis demonstrated a statistically significant difference between *Alu* and SZ patients' Clinical Global Impression Scale (CGI) scores (*P* < 0.05), but no significant differences with abandonment (*P* > 0.05), attack (*P* > 0.05), positive syndrome scale score (*P* > 0.05) and negative syndrome scale score(*P* > 0.05) (Table 3).

**Table 3:** Correlation analysis between *Alu* and scores in patients with psychiatric disorders.

	***Alu*** **(ng/ml)**	
	***r***	***P***
CGI score	0.1638	0.0499
Abandon score	−0.1286	0.1258
Attack score	0.01357	0.8718
Positive syndrome scale totle score	0.038	0.6690
Negtive syndrome scale totle score	−0.1008	0.2555

*P < 0.05 was considered statistically significant*.

*CGI, clinical global impression*.

### The Correlation Between *Alu* and Biochemical Indexes in SZ Patients

We analyzed the correlation between the concentration of *Alu* and biochemical indexes and demonstrated no statistically significant correlation except for RBP (*P* < 0.05), Cyc (*P* < 0.05), and NEFA (*P* < 0.05) (Table 4).

**Table 4:** Correlation analysis between the concentration of the *Alu* and biochemical indexes.

	**Median (interquartile range) (ng/ml)**	***Alu*** **(ng/ml)**	
		***P*** **r**	
Glu (μmol/L)	4.85 (4.48–5.22)	0.2225	−0.1023
CG (mg/L)	1.11 (0.94–1.40)	0.4403	−0.0631
TBil (μmol/L)	9.9 (6.68–14.86)	0.2402	0.0958
DBil (μmol/L)	1.95 (1.2–3.3)	0.2099	0.1023
TP (g/L)	67.2 (64.7–71.43)	0.3524	0.0759
ALB (g/L)	43.45 (40.9–45.8)	0.2217	0.0997
GLO (g/L)	25.1 (22.3–27.1)	0.8788	0.0203
A/G	1.8 (1.6–2)	0.6943	0.0523
ALT (U/L)	19.95 (13.3–36.43)	0.6759	−0.0342
AST (U/L)	21 (17–30)	0.6410	0.0379
AST-m (U/L)	2.8 (2.2–4)	0.1135	−0.2047
GGT (U/L)	17.65 (13.28–26.68)	0.0562	−0.1552
ALP (U/L)	61.77 (52.88–75.89)	0.2949	−0.0855
LDH (U/L)	165 (141–191.5)	0.1404	0.1193
HBDH (U/L)	113.5 (101.3–130.75)	0.7719	0.0376
CK (U/L)	86.7 (62.4–202)	0.3736	0.0724
CK-MB (U/L)	10.3 (8.43–15)	0.0906	0.2168
CHE (U/L)	7818.5 (6742.7–9120.95)	0.2631	−0.0916
PA (mg/L)	259 (229.5–306.5)	0.1086	−0.1311
ADA (U/L)	9.7 (8.25–11.75)	0.8140	−0.0193
BUN (mmol/L)	4 (3.2–4.88)	0.2665	−0.0913
CRE (μmol/L)	60.55 (51.18–73.35)	0.1459	−0.1193
UA (μmol/L)	343.65 (284.88–395.9)	0.1026	0.1338
B2MG (mg/L)	1.33 (1.06–1.62)	0.2869	−0.0875
RBP (mg/L)	34.6 (27.9–40.1)	0.0128	−0.2035
Cyc (mg/L)	0.54 (0.48–0.61)	0.0231	−0.1860
K (mmol/L)	4.06 (3.81–4.28)	0.1855	0.1083
Na (mmol/L)	141.32 (139.68–143.12)	0.5086	0.0542
Cl (mmol/L)	101 (99.11–103.57)	0.4231	0.0657
Ca (mmol/L)	2.29 (2.24–2.35)	0.2692	0.0905
P (mmol/L)	1.24 (1.1–1.37)	0.1033	−0.1331
Mg (mmol/L)	0.83 (0.77–0.88)	0.2439	0.0954
CO_2_ (mmol/L)	24.89 (23.02–27.50)	0.0139	−0.1999
CHOL (mmol/L)	3.98 (3.44–4.64)	0.8987	−0.0120
TG (mmol/L)	1.07 (0.80–1.36)	0.0692	−0.1693
HDL (mmol/L)	1.19 (1.00–1.38)	0.7300	0.0324
LDL (mmol/L)	2.11 (1.76–2.54)	0.7871	0.0253
APOA1 (g/L)	1.09 (0.99–1.20)	0.9840	0.0019
APOB (g/L)	0.73 (0.63–0.87)	0.9392	−0.0072
APOE (mg/L)	40.45 (37.33–45.25)	0.4400	−0.0724
LP(a) (mg/L)	132.1 (63.2–260.35)	0.5501	−0.0561
NEFA (μmol/L)	580.5 (291.53–832.05)	0.0256	0.2072

*P < 0.05 was considered statistically significant*.

### The Value of Serum *Alu* in the Therapy and Progression of SZ Patients

**Figure 4:**
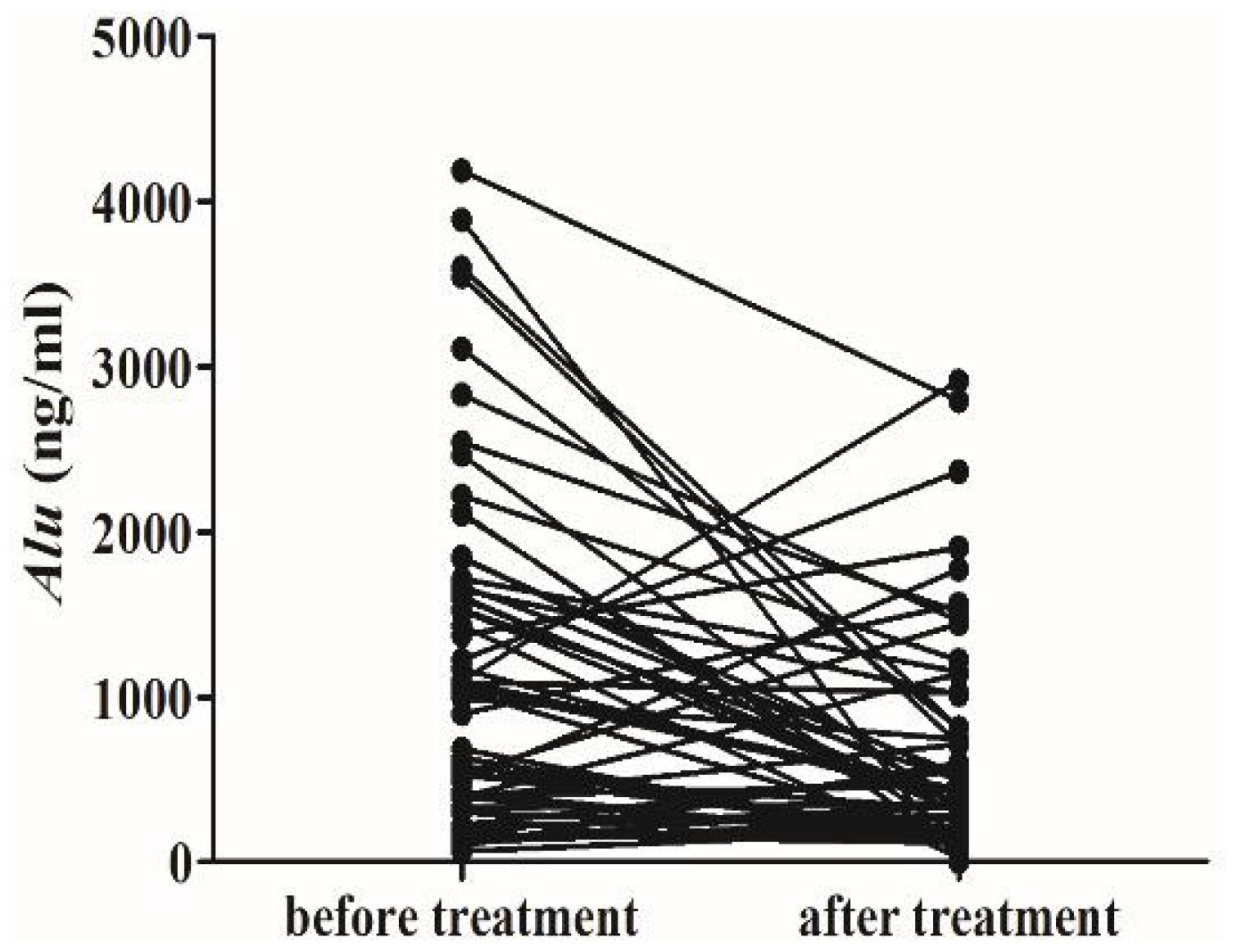
RT-qPCR analysis for predicting cf-DNA as a SZ prognosis biomarker. Line chart of serum cf-DNA levels monitored in the 57 primary SZ patients. The different dots represent the individual subjects and the lines connect the baseline value to the corresponding follow-up.

By trcking the 57 newly diagnosed SZ patients, we found the level of *Alu* before treatment (1040.4 ng/ml, 388.3–1673.4 ng/ml) was higher than the level at post treatment (366.3 ng/ml, 173.3–815.7 ng/ml) (Figure 4). While, the level of *Alu* in the normal control group is (318.3 ng/ml, 253.5–427.4 ng/ml) (*P* > *0.05*).

## Discussion

Recent studies have shown that molecular abnormalities play a role in the development and progression of many mental disorders (28). In this study, we found that the concentration of *Alu* in the SZ group was significantly higher than that of the HC group. Moreover, ROC analysis shows that the *Alu* was helpful in SZ diagnosis. And the concentration of cf-DNA in the post-treatment patients was decreased significantly in 57 newly diagnosed cases.

In this study, we found that SZ patients had higher levels of cf-DNA in their blood than normal controls, which is consistent with the findings of Jiang (29). Environmental or traumatic stress cascades lead to increased the level of *Alu* (30). The *alu*-centric mechanism has been studied to provide a unified framework for many hypotheses regarding the origins of neurodegenerative diseases, including inflammation, oxidative stress, metabolic dysfunction, and protein body accumulation (31). The reactive oxygen species (ROS) emerges from oxygen metabolism, which is the endogenous source of damage. Elevated ROS levels in the brain and peripheral tissues of the patients with SZ triggers a number of cellular responses in order to facilitate apoptosis have been reported (32, 33). Therefore, we speculate that oxidative stress promotes apoptosis, leading to an increase in *Alu* level, and *Alu* level reflects the degree of apoptosis. Our results may provide a new argument for the apoptosis mechanism of schizophrenia and further verify this hypothesis.

A review suggests that an ascent of cf-DNA levels depend on various clinical conditions: lowest cf-DNA levels were found in chronic inflammation, elevated levels in acute inflammation and highest cf-DNA levels were measured in severe infections (34). In our results, the concentration of cf-DNA was not statistically different between undifferentiated, paranoid, and acute SZ-like psychosis. However, the median concentration of cf-DNA in acute SZ-like psychosis was higher than that of the other two subtypes. The different levels of *Alu* of different types suggest that the pathogenesis of different SZ types and the severity of the disease are different, laying the foundation for our later treatment and analysis of the mechanism of different subtypes.

Furthermore, the ROC analysis showed that the AUC between the SZ and normal control group was 0.6787, with a 51.72% sensitivity and 90% specificity. Using ROC to evaluate the diagnostic utility of cf-DNA indicates that serum cf-DNA can effectively distinguish schizophrenia patients from normal people. In this study, the value of AUC is not ideal, and the sensitivity and specificity of cf-DN as a qualified biomarker need to be further improved. Other indicators need to be added to increase the AUC. Therefore, cf-DNA alone cannot be used to diagnose SZ, and other clinical indicators need to be combined. Based on the above-mentioned experimental results, we further analyzed the correlation between cf-DNA and clinical data and laboratory examination results.

Serum *Alu* levels exhibited no significant differences regarding gender, age at onset, disease duration, treatment duration, and treatment plan. This suggests that cf-DNA may be an independent indicator at the cellular level relative to these indicators. Therefore, *Alu* levels will not be affected by gender, age at onset, disease duration, treatment duration, and family history. In addition, there was no statistical difference between *Alu* and SZ patients' abandonment, attack, positive syndrome scale score and negative syndrome scale score, but there was a difference with CGI. In related studies, in order to evaluate the treatment effect of schizophrenia, a relief definition based on the CGI was developed. The CGI score reflects the severity of the disease and the risk of recurrence (35). This result paves the way for further studies regarding the correlation between cf-DNA concentration and disease severity.

We studied the correlation between *Alu* and several biochemical indicators. We found that *Alu* was negatively correlated with RBP and Cyc and positively correlated with NEFA, yet the *r* values were all small, indicating that the correlation was not significant. Although we unexpectedly found a correlation between cf-DNA and RBP, Cyc, and NEFA, the mechanism remains to be elucidated. It may also need to increase the sample size.

We also found that cf-DNA levels underwent dynamic changes after treatment in the primary patients. And the concentration of cf-DNA in the post-treatment patients was decreased. It indicted that the level of *Alu* may be related to the course of the disease. Cf-DNA has been described in various diseases, including inflammation, tumors, trauma, stroke, and myocardial infarction (36–38). And the cf-DNA concentrations are significantly increased in patients with poor outcome (39). It was reported that a decrease in cf-DNA levels in response to therapy and elevations associated with recurrence, suggesting that cf-DNA quantification may facilitate monitoring the disease status (40, 41). Patients with high plasma cf-DNA levels either did not respond to treatment or had a higher risk of recurrence (42). Our result is consistent with the phenomenon reported in the literature.

## Conclusions

Our results show that Alu can effectively distinguish SZ from normal people, and has a certain indicator effect on the prognosis of SZ patients. Further studies should focus on large-scale sample collection and long-term follow-up, adjust for potential confounding factors, and perform an in-depth functional investigation in order to improve the sensitivity and specificity of this potential biomarker. Overall, it may indicate that cf-DNA could improve the diagnosis of schizophrenia and might be a potential target in the antipsychotic treatment of SZ patients.

## Data Availability Statement

The original contributions presented in the study are included in the article/Supplementary Material, further inquiries can be directed to the corresponding author/s.

## Ethics Statement

The studies involving human participants were reviewed and approved by Nantong Forth People's Hospital ethics committee (2019 K007). Written informed consent to participate in this study was provided by the participants' legal guardian/next of kin.

## Author Contributions

L-yC carried out the experiment and wrote the paper. JQ analyzed the date. H-lX performed experiments. X-yL collected samples. S-qJ and Y-jS designed the research. All authors contributed to the article and approved the submitted version.

## Conflict of Interest

The authors declare that the research was conducted in the absence of any commercial or financial relationships that could be construed as a potential conflict of interest.

## Abbreviations

SZ: schizophrenia
AP: Anti-psychotics
AD: Antidepressants
MS: Mood stabilizers
CGI: Clinical Global Impression
Glu: glucose
CG: glycocholic acid
TBil: total bilirubin
DBil: direct bilirubin
TP: total protein
ALB: albumin
GLO: globulin
ALT: alanine aminotransferase
AST: aspartate aminotransferase
GGT: γ-glutamyl transpeptidase
ALP: alkaline phosphatase
LDH: lactic dehydrogenase
HBDH: hydoxybutyrate dehydrogenase
CK: creatine kinase
CK-MB: creatine kinase MB
CHE: pseudocholinesterase
PA: prealbumin
ADA: adenosine deaminase
BUN: urea nitrogen
CRE: creatinine
UA: uric acid
β_2_MG: β_2_-microglobulin
RBP: retinol-binding protein
Cys C: cystatin C
K: kalium
Na: sodium
Cl: chlorinum
Ca: calcium
P: phosphor
Mg: magnesium
CO_2_: carbon dioxide
CHOL: total cholesterol
TG: triacylglycerol
HDL: high density lipoprotein
LDL: low density lipoprotein
APOAI: apolipoprotein AI
APOB: apolipoprotein B
APOE: apolipoprotein E
LP(a): lipoprotein
NEFA: free fatty acid.
